# Sorghum genetic, genomic, and breeding resources

**DOI:** 10.1007/s00425-021-03742-w

**Published:** 2021-11-05

**Authors:** Zhanguo Xin, Mingli Wang, Hugo E. Cuevas, Junping Chen, Melanie Harrison, N. Ace Pugh, Geoffrey Morris

**Affiliations:** 1 Plant Stress and Germplasm Development Unit, Crop Systems Research Laboratory, USDA-ARS, 3810, 4th Street, Lubbock, TX 79424 USA; 2 Plant Genetic Resources Conservation Unit, USDA-ARS, Griffin, GA 30223 USA; 3 Tropical Agriculture Research Station, USDA-ARS, Mayagüez, 00680 Puerto Rico; 4 Crop Quantitative Genomics, Soil and Crop Sciences, Colorado State University, Plant Sciences Building, Fort Collins, CO 80523 USA

## Abstract

**Main conclusion:**

Sorghum research has entered an exciting and fruitful era due to the genetic, genomic, and breeding resources that are now available to researchers and plant breeders.

**Abstract:**

As the world faces the challenges of a rising population and a changing global climate, new agricultural solutions will need to be developed to address the food and fiber needs of the future. To that end, sorghum will be an invaluable crop species as it is a stress-resistant C_4_ plant that is well adapted for semi-arid and arid regions. Sorghum has already remained as a staple food crop in many parts of Africa and Asia and is critically important for animal feed and niche culinary applications in other regions, such as the United States. In addition, sorghum has begun to be developed into a promising feedstock for forage and bioenergy production. Due to this increasing demand for sorghum and its potential to address these needs, the continuous development of powerful community resources is required. These resources include vast collections of sorghum germplasm, high-quality reference genome sequences, sorghum association panels for genome-wide association studies of traits involved in food and bioenergy production, mutant populations for rapid discovery of causative genes for phenotypes relevant to sorghum improvement, gene expression atlas, and online databases that integrate all resources and provide the sorghum community with tools that can be used in breeding and genomic studies. Used in tandem, these valuable resources will ensure that the rate, quality, and collaborative potential of ongoing sorghum improvement efforts is able to rival that of other major crops.

## Introduction

Sorghum (*Sorghum bicolor* L. Moench) is a stress-resilient crop with highly productive NADP-ME type C_4_ photosynthesis and highly efficient nitrogen and water utilization (Wang et al. 2009). It is the fifth-most important cereal crop globally, serving as a staple food for over 500 million people in the arid and semi-arid regions of the world. It is also an increasingly important crop for animal feed, forage, and bioenergy feedstock for production of biofuel and bioproducts. As an annual crop, most sorghum varieties in the temperate region are photoperiod-insensitive and can complete life cycle in about 4 months. With help of winter nursery, two crops can be grown in a year. In greenhouse, it is easy to grow three generations in a year. Sorghum lines from the tropical origin are photoperiod-sensitive and need short days to flower. Sorghum is transformable, but only a few lines, such as RTx430 and P898012, have adequate transformation efficiency (Gurel et al. 2009; Howe et al. 2006; Che et al. 2018). A genotype-independent efficient transformation protocol remains the main obstacle to apply genome editing efficiently in sorghum.

Despite its value, sorghum breeding and genomic studies have lagged other crops like rice and maize. With the completion of a sorghum reference genome sequenced over a decade ago (Paterson et al. 2009), the construction of several sorghum association panels (SAP) (Casa et al. 2008; Morris et al. 2013b; Upadhyaya et al. 2009), the establishment of mutant libraries, the recent completion of sorghum pan-genomes, the availability of sorghum gene expression atlas and the development of sorghumbase online (Addo-Quaye et al. 2018; Jiao et al. 2016; Xin et al. 2008; Tao et al. 2021; Makita et al. 2015; Shakoor et al. 2014), sorghum research has entered an exciting new age. With the vast resources available to producers and researchers, sorghum will become a critical crop for addressing global food and energy security in a changing global climate.

## Resource of induced mutant libraries

A well-categorized mutant library generated from an inbred line with uniform genetic background and detailed annotation of phenotypes provides a powerful resource to isolate independent alleles of mutants with relevant traits. In the past, bulk mutagenesis followed by selection of a phenotype of interest has been very effective in the identification of genes underlying specific phenotypes; however, other mutations or phenotypes, which can be important for processes other than the current interest, are often ignored (Oria et al. 2000; Peters et al. 2009; Porter et al. 1978; Singh and Drolsom 1974). To preserve all mutations for functional genomic studies, Xin et al. designed a systematic approach to develop a pedigreed mutant library (Xin et al. 2008). Individual seeds from an elite inbred line, BTx623, were subjected to chemical mutagenesis by soaking them in various concentrations of ethyl methane sulfonate (EMS) from 0.1 to 0.25% (v/w). BTx623 was chosen for this purpose because it was the sorghum line used to generate the first sorghum reference genome. Next, the mutagenized M_1_ seeds were propagated to the M_3_ generation by single-seed descent. Ten panicles from the M_3_ plants were bulk selected to serve as one pool of M_4_ seeds. Thus, each pool of seeds was derived from a single independently mutagenized M_1_ seed by pedigree. The pedigreed M_4_ seed pools were propagated from M_1_ seeds without selection; therefore, most mutations, including recessive lethal mutations, are preserved in the mutant library. Because the M_4_ seed pools can be replenished by planting and pooling 20 or more panicles from the remaining seeds, the pedigreed mutant library can serve as a permanent resource for screening relevant mutants under a variety of growth conditions. The library now consists of approximately 6400 independent seed pools that can be used to select mutants with traits that are potentially useful for sorghum improvement. A similar approach has been used to generate approximately 10,000 pedigreed seed pools in Purdue University (Addo-Quaye et al. 2017, 2018). Within the pedigreed mutant library, a wide range of phenotypes relevant to sorghum improvement have been observed and the mutants have been selected (Jiao et al. 2016). Furthermore, both forward and reverse genetic resources to efficiently explore the mutant library for traits of potentially useful for sorghum improvement and genomic studies have been developed (Jiao et al. 2016, 2017; Wang et al. 2021). Here, we discuss these resources and how to use them to facilitate sorghum studies. We also discussed a few classes of mutants in detail so that we may demonstrate the utility of the mutant library.

## Fast forward genetics—identify causal mutations from the pedigreed mutant library via bulk segregant analysis and next-generation sequencing

Identification of causal gene mutation underlying a mutant phenotype of interest, termed forward genetic, is instrumental in understanding the mechanisms governing growth and development, signal transduction, and metabolic pathways (http://thearabidopsisbook.org). The conventional method to identify the causal mutation from a mutant is map-based cloning, which requires the analysis of thousands of DNA markers in a large segregating individual F_2_ population (usually over 1000 individual F_2_ plants) to delimit the mutation to a region harboring only a few genes (Jander et al. 2002). The conventional map-based cloning is lengthy and complicated. It often takes a skilled researcher three or more years to identify one gene in model plants, like *Arabidopsis* (Jander et al. 2002).

The rapid innovation in next-generation sequencing (NGS) techniques provides large number of DNA markers at affordable cost (Metzker 2010). Two general approaches have been developed to use the single-nucleotide polymorphic (SNP) markers that are annotated from NGS to map and identify the causal mutations underlying mutant phenotypes of interesting. The first strategy is represented by mapping-by-sequencing (ShoreMap) (Schneeberger et al. 2009; Hartwig et al. 2012) or next-generation map (NGM) (Austin et al. 2011). This approach is very similar to conventional map-based cloning by crossing the mutant to a line that has extensive DNA polymorphism from the mutant. Equal amount of DNA from many homozygous mutants selected from the F_2_ population is pooled and sequenced. The co-segregation of SNP markers with the mutant phenotype is analyzed following the principle of bulk segregant analysis (BSA) (Michelmore et al. 1991). The second strategy is isogenic mapping-by-sequencing, also called MutMap, a variation of the ShoreMap, designed to identify the causal mutations from bulked F_2_ mutants isolated from a cross of the mutant to its original un-mutated parent (Abe et al. 2012; Hartwig et al. 2012; Zhu et al. 2012). If the F_2_ population segregates for a mutant phenotype to the wild-type phenotype at an approximately 1 to 3 ratio, or consistent with recessive mutation, genomic DNA from 50 homozygous of the mutants selected from the F_2_ population is pooled for NGS. The causal mutation for the phenotype is expected to be a 100% of the mutated SNP due to the selection. The mutations that are unlinked with the causal mutation will have a SNP ratio (the number of mutated SNPs/total SNPs) of 0.5, approximately 50% the of the SNPs are the mutated type.

**Fig. 1:**
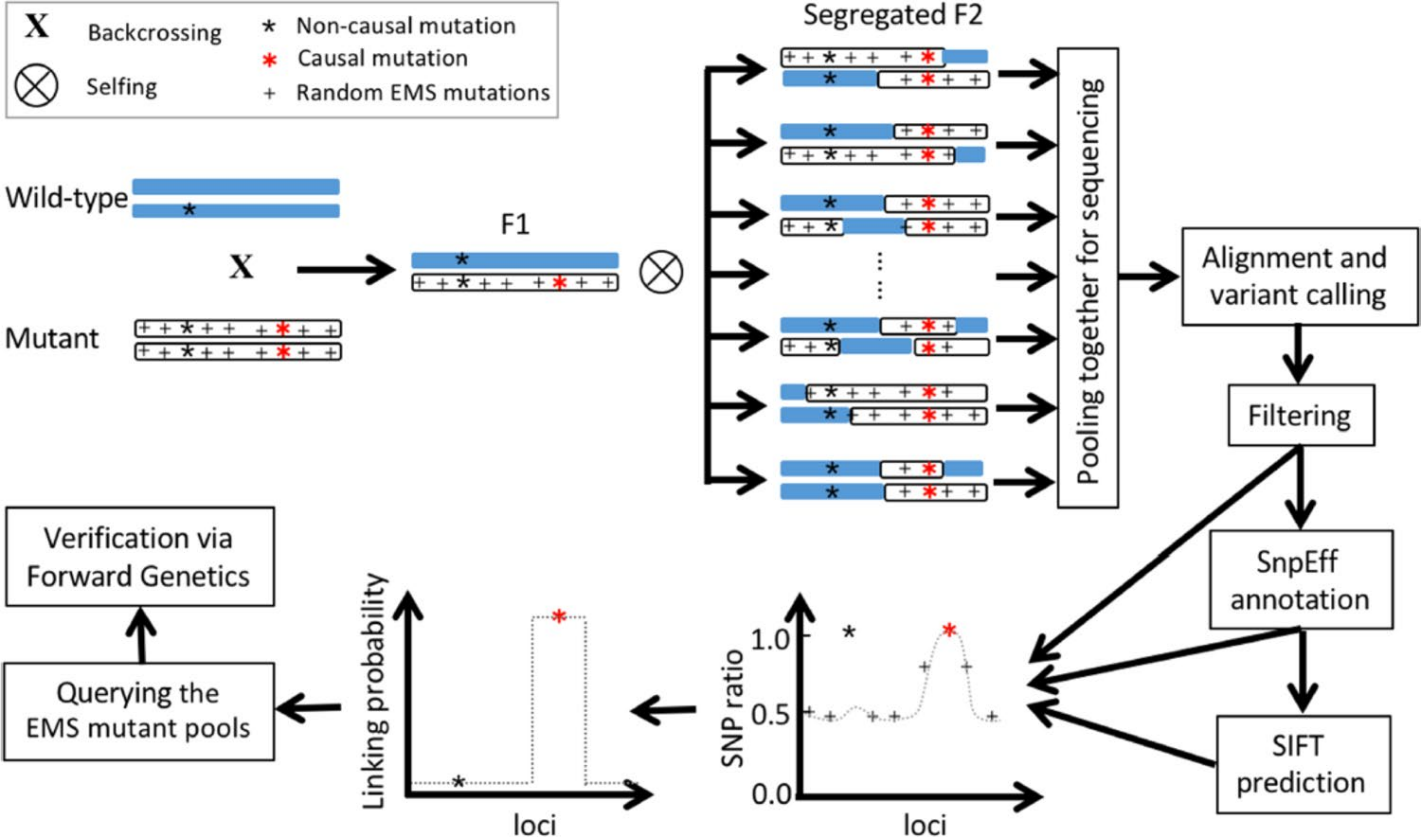
Illustration of the BSAseq Workflow. Creation of the BSAseq mapping population is similar to MutMap. To use the workflow in sorghum, the user only need input the two pair-end sequencing file into the workflow using “Browse” to select the files in the Cyverse Datastore. All other steps are automated. The output is the SNP ratio and linking probability plots, from which, the candidate genes can be selected based on SNP ratio close to 1 in the region with a probability above the cutoff line (the horizontal line in the linking probability plot)

Conceptually, MutMap is a straightforward process; however, in practice, it is often very difficult to distinguish causative mutations from background mutations present in the parental lines before mutagenesis or from sequencing errors in the reference genome, which also show SNP ratios of 1. Furthermore, MutMap requires the SNP ratio to be exactly 1; consequently, the phenotype of the mutant must be 100% correct. To overcome these potential pitfalls in MutMap, Wang et al. (2021) developed a web-based workflow, BSAseq, an interactive and integrated bioinformatic pipeline for identification of causal mutations (Fig. 1). The first step of BSAseq is to cross a mutant of interest to the WT parent. Next, the F_1_ plants are self-pollinated to produce an F_2_ generation. After confirming that the mutant segregation ratio is consistent with a single recessive Mendelian trait at a ratio of approximately 1:3, the genomic DNA is extracted from a pooled sample of 20 homozygous mutants and sequenced to 15 × coverage of the genome by pair-end sequencing on Illuminia platforms. Short reads are aligned to the reference assembly using Bowtie2 (Langmead and Salzberg 2012), and SNPs are called and filtered using Bcftools (Li 2011) to keep the canonical EMS-induced mutations (G → A or C → T) with the desired coverage (e.g., 5–100). SnpEff (Cingolani et al. 2012) is used to annotate and select the mutations with a large predicted effect (missense, nonsense, splice site acceptor, or splice site donor), and SIFT 4G (Vaser et al. 2016) is used to predict whether the mutations are deleterious to the gene. The SIFT score ranges from 0 to 1 and a score of less than 0.05 is generally considered deleterious. These steps, from aligning the short sequences with reference genes to the prediction of deleterious mutations, are integrated into the BSAseq pipeline. All that is required of a user is to input the two sequencing files (pair-ends) into the workflow, and the output of the workflow is the BSA-viewer that consists of two interactive graphs. The first graph is the SNP ratio plot, from which one can identify mutations with a SNP ratio close to 1. Within this plot, there are two types of mutations represented by red or gray circles, wherein the red circles are those with SIFT scores < 0.05 and the gray circles are those with SIFT scores > 0.05, or non-deleterious mutations. The other graph is a linkage probability plot, from which one can determine the probability of the candidate mutation with the predicted SNP ratio occurring by chance. The horizontal line on the plot is the significance threshold for this SNP ratio and can be used to ultimately determine if the SNP ratio is significantly different from the ratio of unlinked SNPs (50%). The possible candidate SNP must be above this threshold to be considered significant. The final and most important criterium is the biological knowledge, i.e., the function of the gene that makes biological sense to the phenotype of interest. To further validate if the mutation is indeed the causative mutation for the phenotype of interest, one can search the gene harboring the putative causative mutation for a second independent allele from the sequenced sorghum mutants (or mutation-indexed mutant library) available on Gramene (http://www.gramene.org/). If the mutant line containing the independent mutation in the same gene also segregates for the phenotype of the mutant of interest, the causative mutations is then confirmed.

Compared with MutMap, the BSAseq requires pooling of only 20 F_2_ mutants, a sequencing depth of 15 × coverage, and is tolerant to the inclusion a few mis-phenotyped F_2_ plants. At current sequencing prices, it takes approximately $200 USD to sequence and analyze one bulked F_2_ population. The BSAseq workflow is publicly available at (https://www.sciapps.org/), and a free account at Cyverse to hold the sequencing data and access the workflow can be obtained. Once the account is activated, the user only needs to upload the two pair-sequencing data to Cyverse Datastore using the free data transfer software Cyberduck (https://cyberduck.io/) or IRODS (https://irods.org/). In addition, an instructional video is available online to help users learn to use the BSAseq workflow (https://www.sciapps.org/). Because there are resources and tutorials available to users, no prior training in bioinformatics techniques is required for a researcher to use this workflow. Although the workflow is designed with data obtained from the sorghum pedigreed mutant library, it can be easily applied to other crops by replacing the sorghum reference genome with the reference genome of the crop from which the mutants are selected.

## Reverse genetics—identification of mutation series from sequenced mutant library

Identification of mutation series in a gene with known sequence to deduce its function by studying the phenotypes of the series mutants is an important genetic research approach called reverse genetics (McCallum et al. 2000a; Till et al. 2003; Wienholds et al. 2003; Gilchrist and Haughn 2005; Winkler et al. 2005; Gilchrist et al. 2006; Xin et al. 2008; Tsai et al. 2011). A technique, dubbed as TILLING (Targeting Induced Local Lesions IN Genomes (TILLING) (McCallum et al. 2000a, b), is usually used to identify the mutant series. The TILLING technique begins with the development of a mutant population by chemical mutagenesis (McCallum et al. 2000b). Genomic DNA is extracted from individual M_2_ plants and pooled by one or multiple dimensions with no more 8 samples per pool. PCR primers are designed to cover the regions of interest. After the PCR amplification of the targeted regions, the heteroduplex formation through denaturation and re-annealing, the mutation within the amplified region is detected through cleavage of the heteroduplex by the endonucleases that can recognize single base pair mismatch, such as CelI (Till et al. 2003). In TILLING, each pair of primers requires meticulous optimization. Thus, it can be cost-prohibitive to analyze a large number of genes.

**Fig. 2:**
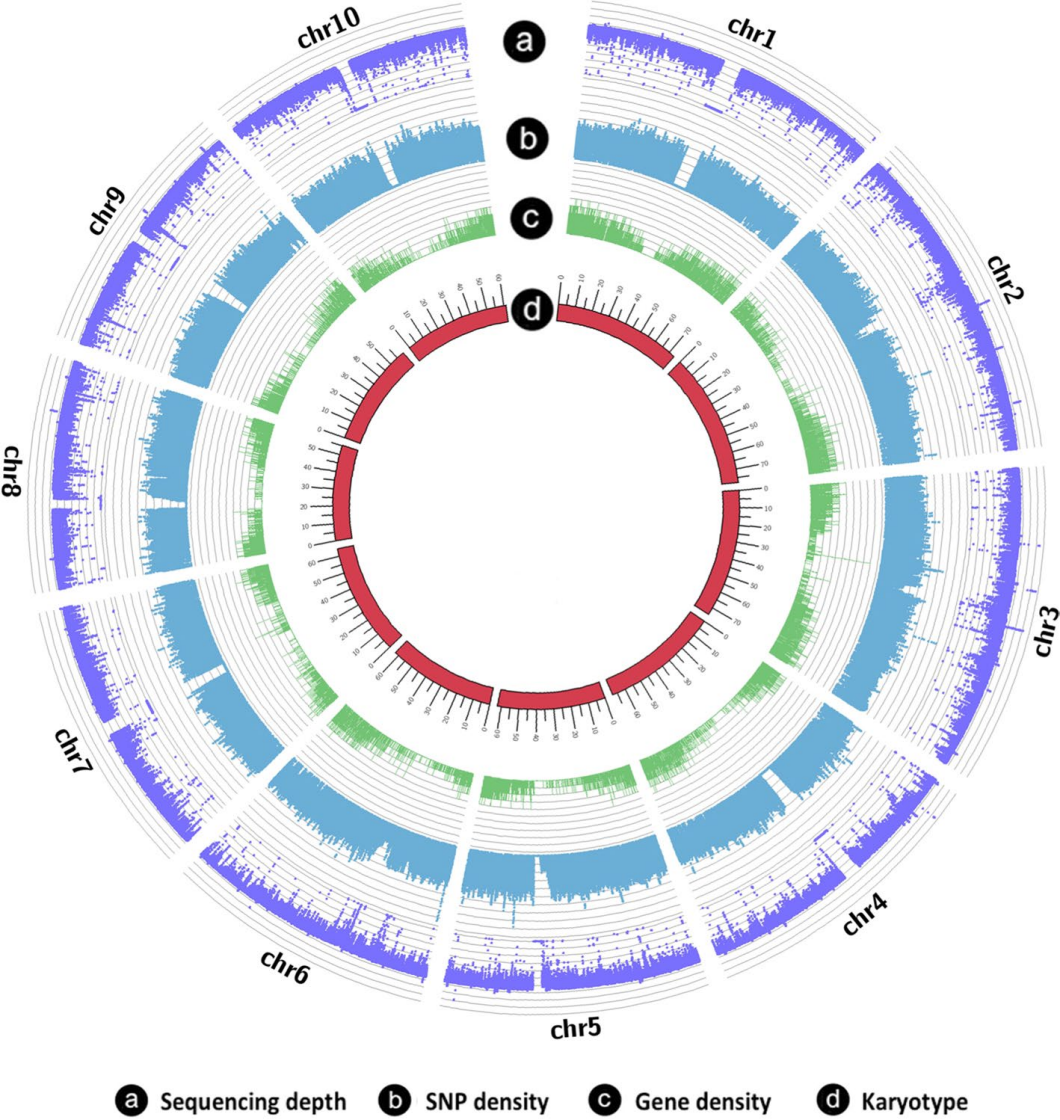
Distribution of EMS-induced mutations. The circle b is the distribution of the EMS-induced mutations (SNPs), which evenly cover the 10 chromosomes except the centromere regions

Due to the increased output of high-quality DNA sequences and the decreased price of next-generation sequencing technologies, it is possible to conduct whole-genome sequencing of the pedigreed mutants as a resource for reverse genetics by searching gene mutations online. Jiao et al. (2016) sequenced 256 mutant lines to an average coverage of 16 × of the genome, an effort that produced 1.8 million EMS-induced mutations (i.e., G → C or A → T mutations). The average mutation rate is 11.2/Mb, or about one SNP per 344 bp in the 256 lines. All 10 chromosomes were evenly covered with mutations (Fig. 2). About 236,000 (6.2%) of the SNPs are in 30,294 genes, which covers about 92% of the genes in the sorghum genome (Table 1). A total of 111,850 non-synonymous SNPs are in the exons of 25,605 (77.5%) genes, with an average of 4 mutations per gene. There were 8043 stop-gain or splice junction mutations that potentially produce a knockout mutation of the genes. Addo-Quaye et al (2018) sequenced 586 mutants and discovered 1,275,872 homozygous and 477,531 heterozygous EMS-induced mutations (Addo-Quaye et al. 2017, 2018). There are 56,514 homozygous missense mutations in 23,227 genes, among which 4035 are high-impact homozygous mutations in 3637 genes, and each of these sequencing results can be searched on Gramene (http://www.gramene.org/). A collaboration is currently underway with Joint Genome Institute (JGI) of the Department of Energy to sequence additional 1000 pedigreed mutant lines. Based on two published sequencing results, we would obtain a total of > 10 million canonical EMS-induced mutations after completion of the sequencing of additional 1000 lines. More importantly, we would obtain over 150,000 deleterious mutations (SIFT score < 0.05), including > 35,000 knockout mutations (stop-gained and splice junction mutations). With these collections of sequenced mutants, most genes in the sorghum genome would have knockout or deleterious mutations, making the sequenced data a useful resource for reverse genetics; Moreover, these collections could allow for the validation of candidate genes in biparental and genome-wide association mapping of QTLs of important agronomic traits and for the identification of additional independent alleles for genes predicted from the BSAseq.

**Table 1:** Annotation of mutations

Effect type	SNPs	Genes
UTR 3′	44,120	18,772
Start gained	5237	4326
UTR 5′	29,452	14,055
Non-synonymous coding	86,070	25,311
Start lost	136	136
Stop gained	4652	4043
Synonymous coding	138,416	22,035
Splice site acceptor	862	834
Splice site donor	636	621
Splice site region	6894	5260
Intron	195,916	21,443
Upstream 5 kb	480,763	31,358
Downstream 5 kb	464,091	31,141
Intergenic	1,457,491	–

The EMS-induced mutations (SNPs) were classified according to their location and potential effect on gene expression and protein structure

## New dwarf mutants

The semi-dwarf trait is generally considered the single most impactful trait in the development of modern high yielding crops. The wave of adoption of semi-dwarf genes in plant breeding that was coupled with increased usage of irrigation and chemical fertilizers, a period known as the “Green Revolution”, dramatically increased world grain production and successfully averted a catastrophic food shortage that was predicted would occur (Hedden 2003). Unlike wheat (*Triticum aestivum* L.) and rice (*Oryza sativa* L.), which require only a single dwarf locus to breed for semi-dwarf plants (Peng et al. 1999; Sasaki et al. 2002; Wang et al. 2017), sorghum requires multiple dwarf loci stacked together to breed semi-dwarf plants because any single locus is insufficient to create the desired reduction in height (Quinby and Karper 1954). All four known dwarf loci used since the beginning of breeding semi-dwarf sorghum can be traced back to the same sources described in the early 1950s (Quinby 1975; Quinby and Karper 1954), and the causal gene mutations for three of the four dwarf loci have since been identified. The *dw3* locus encodes P-glycoprotein modulating auxin polar transport (Multani et al. 2003). The *dw1* locus encodes a novel protein (Hilley et al. 2016; Yamaguchi et al. 2016). It has recently been suggested that *dw1* may play a role in the brassinosteroid signaling pathway (Hirano et al. 2017). The *dw2* locus encodes a protein kinase, but the signal pathway in which the *DW2* gene functions is not known (Hilley et al. 2017). The gene corresponding to the *dw4* locus has not been identified at the time of this writing. In addition to the four known dwarf loci, other dwarf loci or modifying loci are also inadvertently selected during the process of sorghum improvement (Hashimoto et al. 2021). An in-depth analysis of plant height variations in the SAP with known dwarf loci as covariates shows that at least 10 loci are involved in sorghum height variation (Li et al. 2015). At this time, none of the dwarf loci currently used in sorghum breeding are mapped near the canonical Green Revolution dwarf genes that have been identified in other cereal crops (Wang et al. 2017).

To make semi-dwarf hybrids, both seed parent and pollinator parents must have the same set of dwarf genes, which makes it likely that both parents may carry the same genome segments linked to the dwarf loci. There are strong linkages between the dwarf loci and various maturity loci that are required for flowering in temperate regions, which can make it difficult to utilize dwarf genes in sorghum. The large scale conversion of exotic germplasm from Africa into semi-dwarf and early maturing (photoperiod-insensitive) lines, known as the Sorghum Conversion Program, created thousands of new lines with unique traits and provided the sorghum industry with a large supply of raw breeding material (Stephens et al. 1967). The efficiency of the Sorghum Conversion Program is retrospectively evaluated with over 50,000 SNP markers for 800 pairs of converted lines and their original exotic parents (Thurber et al. 2013). BTx406, a four-dwarf line, is the donor parent for dwarf and maturity loci in most of the Sorghum Conversion Program. One surprising discovery from this retrospective analysis is that large segments of chromosomes 1, 2, 9, 10, and nearly all of the markers on chromosome 6 are consistent with the donor parent BTx406 (Thurber et al. 2013). These homozygous regions may be present in most sorghum hybrids, and this could be a major contributing factor to the prevention of full expression of hybrid vigor in sorghum.

Researchers and plant breeders may wonder why improvement in sorghum requires pyramiding three or more recessive dwarf loci to breed semi-dwarf varieties while wheat and rice only require a single locus to bring plants to their desired height. One hypothesis to explain this key difference could be that researchers have not yet found the optimal dwarf gene for breeding dwarf sorghum. As noted earlier, none of the dwarf genes that have been identified in sorghum appear to be involved in gibberellic acid (GA) signaling or biosynthesis, while the critical genes in other cereal crops are involved in these processes. Since none of the currently known dwarf genes can singularly reduce the height of sorghum to desired levels, breeders must stack multiple dwarf loci until the plant reaches the desired height for combine harvesting. Unfortunately, pyramiding multiple recessive dwarf genes in sorghum is complicated, expensive, and introduces the possibility of reduced hybrid vigor.

**Fig. 3:**
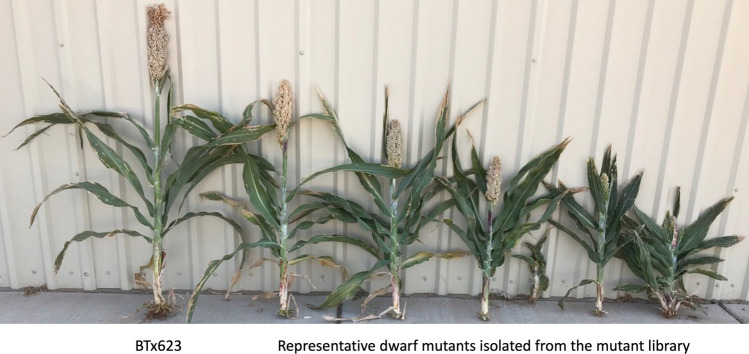
Phenotypes of a few dwarf mutants identified from the sorghum pedigreed mutant library. The wild-type BTx623 carries three homozygous conventional dwarf loci *dw1*, *dw3*,* dw4*. The new dwarf mutants carry a novel dwarf locus in addition to the three conventional loci

We have initiated a systematic approach to identify superior dwarf mutations that can be used alone to breed semi-dwarf sorghum varieties. Dwarf mutant phenotypes are frequently observed in the mutant library; indeed, from approximately 6400 pedigreed lines, over 300 independent dwarf mutants have been isolated (Fig. 3). Some of the dwarf mutants exhibit valuable agronomic traits, such as large panicles and increased seed size, and we are currently in the process of identifying the causative mutations for these dwarf mutants. The genes involved in GA signaling or biosynthesis and the genes identified from mutants that have good agronomic traits will be prioritized for further characterization. We will introduce these mutations into tall sorghum lines that currently have no dwarf genes to determine if any of these mutations can be used to reduce resulting sorghum hybrids to a desirable height without needing to stack multiple dwarf loci.

Several mutations in genes involved GA signaling or biosynthesis were identified from 256 sequenced lines; however, they either have no immediately apparent effect on height or are too short to be considered useful for breeding purposes. Fortunately, efforts are underway to sequence an additional 1000 mutant lines, which may reveal leaky mutations in these genes which can alter function enough to achieve a more ideal plant height.

## Mutants with altered tolerance to high temperature

High-temperature (HT) stress negatively impacts crop yield worldwide. It is urgent to enhance HT tolerance in crops due to the escalated trend of global warming. It has been well established in model plants that HT has many negative impacts on plants (Burke and Chen 2006; Iba 2002; Kotak et al. 2007; Larkindale et al. 2005; Djanaguiraman et al. 2014). Mechanisms of HT tolerance discovered from model organisms include heat shock proteins (HSPs) (Gurley 2000; Hong and Vierling 2000; Nieto-Sotelo et al. 2002), membrane stability under HT through adjustments of membrane lipid composition and fatty acid unsaturation levels (Alfonso et al. 2001; Chen et al. 2006b; Falcone et al. 2004; Marcum 1998; Sung et al. 2003), and production of antioxidants (Burke and Chen 2006; Chen et al. 2006a; Larkindale and Huang 2004; Wang et al. 2006).

Crops under field conditions often experience a combination of HT and drought stresses. Little is known about the mechanisms of how field crops cope with HT stress, probably more complex than that observed from model organisms under controlled laboratory conditions (Chen et al. 2012). Chen et al. (2010) observed that all known mechanisms of HT tolerance, such as expression of heat shock proteins, are very similar between two maize lines with contrast HT tolerance in the field under both normal or HT stress. Instead, the two maize lines differ significantly in phosphatidic acid (PA), a minor phospholipid acting as a signal molecule in plants, before or after a high-temperature stress (Chen et al. 2010).

The hot summer seasons that often occur in Lubbock, Texas provide an appropriate environment to evaluate HT injury in maize. Maize plants display a diverse HT injury phenotype from leaf firing (death of leaves), tassel blast, to reduction in pollen production (Chen et al. 2012). However, sorghum, one of the most HT tolerant crops, shows little damage under similar environmental conditions (Chen et al. 2017). Thus, sorghum could serve as an excellent source for isolating HT tolerant genes that can subsequently be used to improve HT tolerance in crops prone to HT injury.

**Fig. 4:**
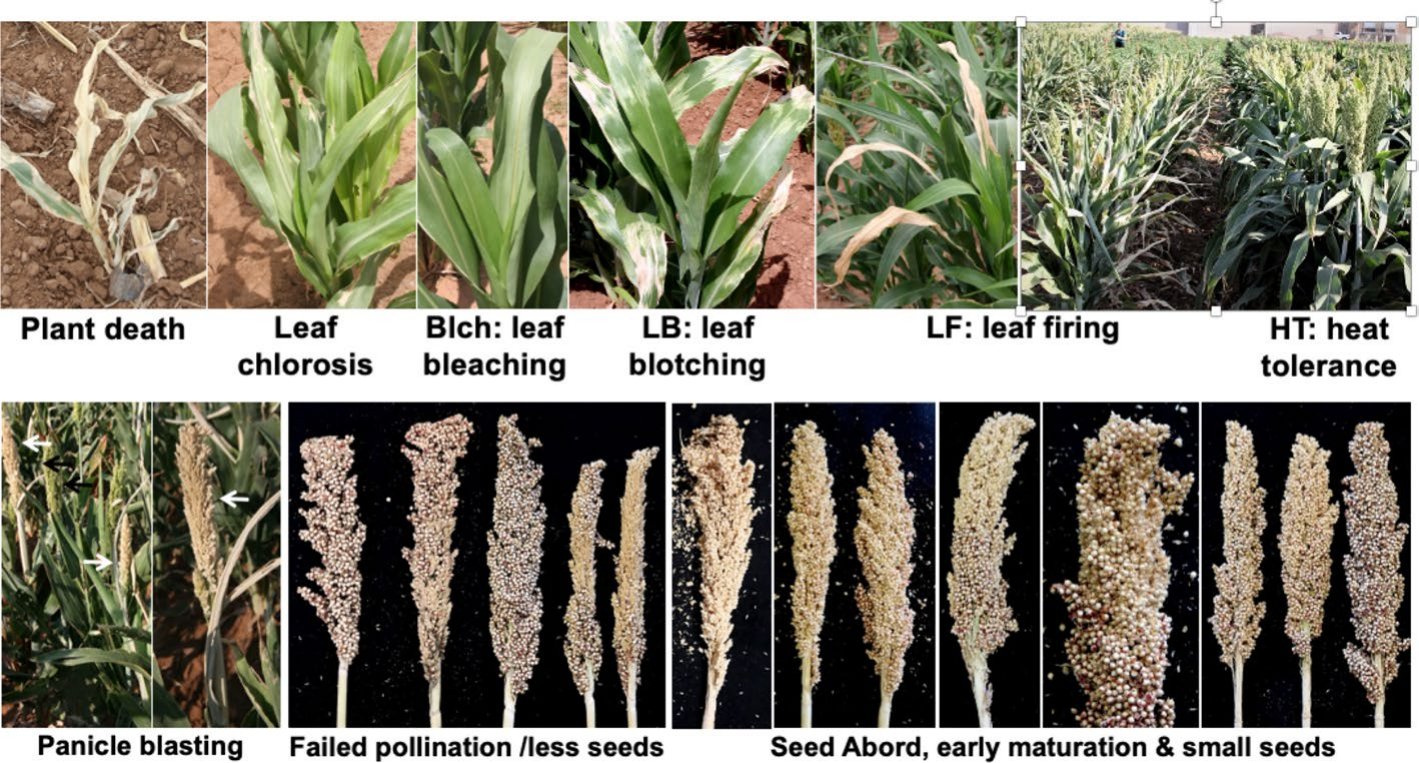
Various high-temperature-sensitive phenotypes observed from the mutant library. The top panel shows the high-temperature-sensitive phenotypes during vegetative stage. The bottom panel shows the phenotypes during reproductive stage

The sorghum mutant library has displayed a variety of HT injury phenotypes that nearly phenocopy the heat-sensitive phenotypes observed in field grown maize (Chen et al. 2012). These phenotypes include plant death, leaf rolling, leaf bleach/blotch, leaf firing, panicle blasting, reduction in pollen production, and a reduction in seed set and seed size (Fig. 4). Because maize and sorghum share a common ancestor as recently as 11 million years ago (Paterson et al. 2004), the high-temperature-sensitive mutants may serve as tools to dissect the mechanism of high-temperature tolerance of maize under field conditions. For example, if a heat-sensitive mutation in sorghum is mapped close to high-temperature tolerance quantitative trait loci (QTL) in maize, the sorghum mutant may be used to clone the sorghum gene through bulked segregant analysis coupled with next-generation sequencing (BSAseq) (Wang et al. 2021). Once a causative mutation/gene is identified, a natural allele of the gene with enhanced HT tolerance may be searched from the vast collection of sorghum germplasm for improving HT tolerance in sorghum. Similarly, the causative gene(s) can be overexpressed in maize or other crops to improve their HT tolerance.

## Multi-seeded mutants

**Fig. 5:**
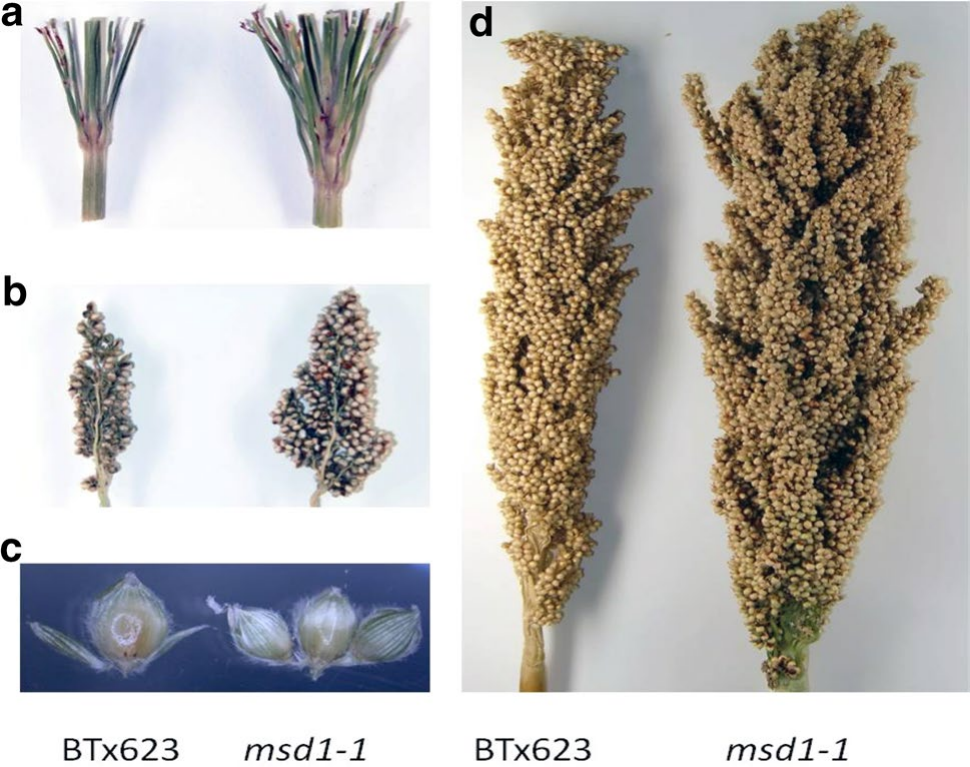
Panicle architecture of a *msd* mutant. The *msd* mutants has increased size and numbers of primary branches. In addition, both sessile and pedicellate spikelets are fertile and capable of producing seeds

Grain yield per acre is determined by the number of plants per acre, the number of effective tillers per plant, the number of grains per panicle, and the grain weight, which is usually expressed as the weight of a thousand grains. Grain number per panicle is a major determinant of grain yield in sorghum and other cereal crops (Saeed et al. 1986; Ashikari et al. 2005; Duggan et al. 2000; Reynolds et al. 2009; Richards 2000). The sorghum panicle bears many primary branches, upon which several secondary branches can develop. Sometimes, tertiary branches can even develop from the secondary branches (Brown et al. 2006; Burow et al. 2014). Sorghum panicles produce two types of spikelets: the sessile spikelets that are directly attached to flower branches and the pedicellate spikelets that are attached to flower branches through a short petiole called a pedicel (Walters and Keil 1988). In the wild-type BTx623 and other natural accessions of sorghum, the sessile spikelets bear hermaphrodite flowers with both female and male floral organs that will eventually develop into seeds (Fig. 5). The pedicellate spikelets are either sterile or staminate and will eventually abort before a seed is produced (Karper and Stephens 1936).

We have isolated and characterized a novel class of sorghum mutants, referred to as multi-seeded (*msd*) mutants, in which the developmental arrest of the pedicellate spikelets does not occur. In the *msd* mutants, all sessile or pedicellate spikelets develop hermaphrodite flowers and could produce seeds. In addition, the *msd* mutants have shown an increased number and length of the primary inflorescence branches. Thus, the *msd* mutants have the potential to produce up to double the grain number per panicle as compared to the wild-type BTx623 (Burow et al. 2014; Jiao et al. 2018). Over 100 independent *msd* mutants have been isolated from the mutant library, and incomplete genetic analyses indicated that these *msd* mutants represent at least 8 complement groups of recessive mutations. In addition, we have isolated several *msd* mutations that are dominant, although further investigation will be required to confirm this and explore its use in sorghum improvement.

Three *MSD* genes have been identified by BSAseq (Wang et al. 2021). The *MSD1* gene (SORBI_3007G135700) encodes the TCP (Teosinte Branched/Cycloidea/proliferating cell nuclear antigen factor) transcription factor, which can activate several enzymes in the jasmonic acid (JA) biosynthetic pathway (Jiao et al. 2018). The *MSD2* gene (SORBI_3006G095600) encodes 13-lipoxygenase (LOX), which catalyzes the conversion of free linolenic acid (18:3, a fatty acid with 18-carbon chain and three double bonds) to hydroperoxy octadecadienoic acids (HPODE), which is the first step of JA biosynthesis (Gladman et al. 2019). The *MSD3* gene (Sobic.3001G407600) encodes a major linoleic (18:2) desaturase FAD7, which is required for biosynthesis of linolenic acid, the substrate for JA biosynthesis (Dampanaboina et al. 2019). Based on the three *MSD* genes identified, it appears that JA plays an important role in the development of the pedicellate spikelets. Both male and female floral organs initiate normally in the pedicellate spikelets of the wild-type BTx623 and the *msd* mutants (Jiao et al. 2018). These floral organs are subsequently aborted in the wild-type pedicellate spikelets but continue to develop in the *msd* mutants. The current hypothesis is that a developmental signal in the pedicellate spikelets activates *MSD1* shortly after floral organ initiation. Then, the MSD1 triggers the burst production of JA that ultimately leads to the abortion of the floral organs through JA-mediated programmed cell death. In the pedicellate spikelets of the *msd* mutants, the lack of this burst production of JA cannot activate the programed cell death pathways, which results in floral organs that continue development into fertile flowers and grains. It is currently unclear whether other processes are involved in the development of the pedicellate spikelets; however, characterization of the other *msd* mutants will likely uncover these processes.

## Other mutants

The mutant library contains many other classes of mutants that cannot be fully described in this review. The mutants with altered agronomic traits are listed in Table 2. One class of mutants with erected leaf (*erl*) architecture particularly worth mentioning because the erect leaf architecture in maize has been considered as an important trait leading to the eightfold yield gains after the Green Revolution. Duvick and Cassman (1999) compared 10 morphological and agronomical traits in 36 maize hybrids released from 1936 to 1991 and found that leaf angle score of the new hybrids displayed an improvement of 122% over the old ones (Duvick and Cassman 1999). The modern maize hybrids with more acute (erect) leaf angle can be planted at higher density to capture more solar radiation per unit land area (Duvick and Cassman 1999). Erect leaf mutants in rice is also considered as an important trait to increase biomass and grain yield (Sakamoto et al. 2006).

**Table 2:** A selection of mutants observed from the sorghum pedigreed mutant library

Mutants	Number of lines	Potential application of the trait
Erect leaf (*erl*)	23	Biomass and grain yield
Brown midrib (*bmr*)	30	Biomass conversion efficiency
Multiple tiller (*mtl*)	120	Biomass yield
Early flowering (*efl)*	7	Biomass and grain yield
Late flowering	5	Biomass yield
Stiff stem (*stf*)	13	Biomass and grain yield
Early senescence (*esn*)	7	Early dehydration of biomass
Multiple seed (*msd*)	> 100	Grain yield
Dominant msd (*msd/d*)	6	Grain yield
Large seed (*lsd*)	18	Grain quality
bloomless (*blm*)	107	Water use efficiency
Heat sensitive (*hs*)	> 200	Tolerance to high temperature
Nuclear male-sterile	> 200	Develop new hybrid breeding system
Dwarf (*dwf*)	> 300	New super dwarf to improve hybrid vigor

Updated from Jiao et al. (2016)

Compared with modern maize hybrids, sorghum exhibits an open canopy with wide leaf angles that are nearly parallel to the ground. The only reported sorghum mutant that has erect leaf angle is the ligule-less mutant (Singh and Drolsom 1973). Due to the presence of other undesirable agronomic characteristics, this mutant has not been used to improve leaf angle in sorghum breeding programs.

To identify new genetic resources for improving leaf angle, we designed a systematic approach to search for sorghum mutants with erect leaves. Among the 6,400 M_3_ plots in the field, over 50 plots segregated for leaf angles that differ from the wild-type BTx623 (Xin et al. 2009). Eleven of these mutants were confirmed in the next generation (M_4_) and several mutants have similar or slightly larger panicles as compared to the wild type. Most of the erect leaf mutants are caused by a recessive mutation on a single nuclear gene, and efforts are currently underway to identify the causative mutations for the erect leaf mutants. Once the causative mutations are identified, it should not be difficult to breed the erect leaf phenotype into elite sorghum lines using either marker-assisted backcrossing or genome editing methodologies.

We have also isolated novel brown midrib (*bmr*) mutants that can be used to improve digestibility of sorghum stalks, bloomless (*blm*) mutants that can be used to dissect the mechanisms of drought tolerance and high water use efficiency of sorghum, mutants with altered root morphology, nuclear male-sterile mutants for developing a two-line breeding system, and other mutants that could be useful in future studies (Chen et al. 2019a; Jiao et al. 2017; Saballos et al. 2012; Sattler et al. 2014; Scully et al. 2016; Tishchenko et al. 2020; Xin et al. 2017). The mutant library may possess many other valuable mutations that could eventually be identified and exploited to introduce novel traits for genomic studies and breeding.

## Genome editing and mutagenesis

Since the publication of a seminal study using the Clustered Regularly Interspaced Short Palindromic Repeats (CRISPR)/CRISPR-associated (Cas) systems to induce mutations in genomes at precise locations, genome editing technology has advanced rapidly (Jinek et al. 2012). With these improved genome editing technologies, many mutations of any type can be introduced into genomes at pre-determined positions simultaneously (Anzalone et al. 2020, 2019; Zuo et al. 2019). Genome-editing technologies promise to transform breeding in both animals and plants (Chen et al. 2019b). The first genome editing in sorghum was performed on immature embryos using CRISPR/Cas9/sgRNA-mediated targeted gene modification but did not produce stably-edited plants (Jiang et al. 2013). Afterward, several studies of genome editing have been reported on sorghum (Li et al. 2018; Char et al. 2020; Liu et al. 2019; Che et al. 2018). Although transformation techniques have been developed to apply genome-editing in a few sorghum lines, only RTx430 and P898012 have reasonable transformation efficiency (Gurel et al. 2012; Liu and Godwin 2012). Development of genotype-independent transformation methods remains the primary obstacle to applying genome editing technologies in sorghum.

Although genome-editing tools can almost introduce any type of mutations in genomes at precise locations, it is critical to know the target gene to be edited, or ideally, the causal mutations in the gene to achieve successful outcome quickly. With the exciting development of improved editing technologies and new ways to apply these technologies that circumvent tissue culture, it is still a challenge to edit all genes in a genome in multiple alterations to determine the impact of these alterations on plant health, productivity, and quality (Wang et al. 2021). On the other hand, mutant libraries derived from induced mutagenesis provide efficient resources to identify the causal mutations through reverse and forward genetics. Given each mutant line carrying thousands of induced mutations (Jiao et al. 2016), the desired traits can be obtained by screening a limited number of mutant lines and in a reasonable time frame (Addo-Quaye et al. 2018; Jiao et al. 2016). The causal mutation underlying the traits of interesting can be identified with BSAseq at a cost less than $200 in sorghum. Although the BSAseq is developed in sorghum, it can be used in other crops by simply changing the reference genome (Wang et al. 2021). We anticipate that BSAseq will become even more affordable for identifying causative mutations, providing promising targets for genome editing. However, induced mutant populations often have a high density of background mutations (Addo-Quaye et al. 2018; Jiao et al. 2016), which must be removed before the mutants are used in breeding. Although recurrent backcrosses can remove unlinked background mutations, it will take several generations to remove 90% of the unlinked mutations. Furthermore, no effective genetic method currently exists for removing the linked mutations. On the other hand, genome editing can introduce precise mutations with few or no offsite mutations, allowing rapid introduction of superior traits into elite germplasm for breeding. The combination of affordable and fast target gene discovery using BSAseq with precise genome editing will truly revolutionize plant breeding.

## Genome sequences

Genome sequences are an important resource for understanding gene contents and functions and are critical to many genomic studies. The first sorghum genome sequence was completed in an elite grain sorghum inbred line, BTx623, over 12 years ago (Paterson et al. 2009). This genome has served as the reference or “blueprint” for genome-wide analyses to identify genetic variations from other sorghum accessions. For comparative genomics, two sweet sorghums (Keller and E-tian) and one grain sorghum (Ji2731) were sequenced using the whole-genome shotgun strategy and Illumina Genome Analyzer sequencing technology and compared with BTx623 by a group of scientists in China (Zheng et al. 2011). A large number of genetic variations were identified among these four genomes including 1,057,018 single-nucleotide polymorphisms (SNPs), 99,948 insertion/deletions (Indels), 16,487 present/absent variations (PAVs), and 17,111 copy number variations (CNVs). Variations in 1442 genes were identified between the sweet and grain sorghums. Sweet sorghums are enriched with genes related to starch and sucrose metabolism as well as lignin- and coumarine-biosynthesis pathways. Subsequently, 44 sorghum accessions, including a wild relative (*Sorghum propinquum*) were sequenced (Mace et al. 2013b). This effort identified untapped genetic potential in sorghum accessions indigenous to the African continent.

Ten years after the first sorghum reference genome was published, a high-quality reference genome sequence (version 3) was assembled with an integrated approach of deep sequencing, genetic linkage analysis, and transcriptome data. Compared with the original reference genome, the new reference genome size added 29.6 Mb, the number of annotated genes increased 24% to 34,211, and the sequencing error rate was reduced tenfold to 1 per 100 kbp (McCormick et al. 2018). Thanks to the development of new technologies, a chromosome-level de novo assembly was generated from a sorghum inbred line, Tx430 (Deschamps et al. 2018). In this genome assembly, 90% of the genome was covered with merely 29 scaffolds. A chromosome-level reference genome has also been generated in a sweet sorghum Rio (Cooper et al. 2019). Despite the apparent difference in agronomic traits between sweet and grain sorghums, the genomes are highly similar in structure (Cooper et al. 2019). Comparative transcriptomics has indicated that the variation in expression of genes related to high stalk sugar content, changes in the activity and localization of transporters, and the timing of sugar metabolism all may play a critical role in sweet sorghum.

Compared with high-quality genome sequencing from individual lines, pan-genome sequences, which consist of genome sequences assembled de novo from many representative genetic diverse lines, can capture the full genomic diversity of a species. Tao et al. (2021) performed a de novo assembly of 13 diverse lines representing the cultivated sorghum and its wild relatives. Along with the publicly available genome sequences, 16 genome sequences were used to construct the sorghum pan-genome sequence. This pan-genome sequence has 955 Mb and contains 44,079 gene families. When the other 15 genome assemblies were anchored to the reference BTx623 genome, 0.3 to 0.5 million insertions/deletions and 15,293,465 SNPs were identified. Gene copy number variations were also identified in 429 and 1118 genes. Surprisingly, 64% of gene families show presence/absence variation (PAV) among genomes. Within this sorghum pan-genome sequence, the genes are classified as either core genes that are present in at least 15 genomes, shell genes that are present in 2–14 genomes, or cloud genes that are present in only one genome. In sorghum, core genes only represent 36% (15,867), shell genes represent 63.6% (28,026), and cloud genes represent 0.4% (186) of the total gene families. The proportion of dispensable genes (shell plus cloud) in the sorghum pan-genome is much higher than that reported in *Oryza sativa* (54%), *Glycine max* (49%) and *Brachypodium distachyon* (45%), indicating that sorghum may have greater genetic diversity.

RNA-seq data and functional enrichment analyses indicate that core genes are enriched in basic and critical functions (such as developing process, RNA processing, reproductive system development, leaf and seed development, cell differentiation, and chloroplast organization), while dispensable genes are enriched in adaptive biological processes (such as secondary metabolic processes, cellular metabolic processes, and amino acid transport). The pan-genome sequence provides the sorghum community a solid genetic foundation for understanding trait biology and a platform for mining genetic diversity for future sorghum improvement and gene discovery.

## Sorghum germplasm

### World collections and gaps

Sorghum has a rich worldwide collection of conserved germplasm, with at least 20 sorghum gene banks in the world, among which are four major centers. The International Crops Research Institute for the Semi-Arid Tropics (ICRISAT) in India holds 37,949 accessions collected from 92 countries (ICRISAT 2021). The United States Department of Agriculture (USDA) National Plant Germplasm System (NPGS) maintains over 45,000 accessions at the Plant Genetic Resources Conservation Unit in Griffin, GA (USDA 2021). The Institute of Crop Science, Chinese Academy of Agricultural Sciences (ICS-CAAS), China, holds 18,263 accessions. The National Bureau of Plant Genetic Resources (NBPGR) of India holds 20,221 accessions (NBPGR 2021). In total, over 240,000 accessions are safeguarded in ex-situ gene banks. Cultivated accessions and wild weedy relatives account for 98.3% and 1.7% of the collection, respectively (Upadhyaya et al. 2016). These collections may have redundancies because of the frequency of exchange among the gene banks; however, the level of this redundancy has yet to be fully understood. To increase the efficiency of conservation and utilization, the redundant accessions must be identified in future studies.

Although many sorghum accessions have been collected and deposited in the world gene banks, geographical and taxonomic gaps still exist. Among 6415 accessions from West and Central Africa (WCA) analyzed with passport and characterization data, clear geographical gaps were identified in Burkina Faso and Nigeria. In addition to these geographical gaps, taxonomic gaps also existed. For example, 22 species within the sorghum genus have been reported, but the WCA collection has only three species (Upadhyaya et al. 2017a). Geographical and taxonomic gaps were also identified at the ICRISAT sorghum gene bank from the South Asia collection (Upadhyaya et al. 2017b). Within the sorghum genus, there are five subgenera. *Sorghum bicolor* belongs to the subgenera of *Eu Sorghum*, within which there are three species: *Sorghum bicolor*, *Sorghum halepense* (Johnson grass), and *Sorghum propinquum*. Within the *S. bicolor* species, there are three subspecies: *subsp. bicolor*, *subsp. verticilliflorum*, and *subsp. drummondii* (Sudan grass). The *subsp. bicolor* has five races (bicolor, caudatum, durra, guinea, and kafir) and ten intermediate races (Ananda et al. 2020; Harlan and Wet 1972; Lazarides et al. 1991). There are about 24 species within the *Sorghum* genus, 17 of which are found in Australia. As with other major cereal crops, use of the sorghum wild relatives (CWR) in breeding is a promising approach for enhancing the genetic diversity and sorghum yield (Mammadov et al. 2018). Due to human activities and their impacts, such as urbanization, cultivation of new varieties, and climate change, the habitats of some sorghum wild species are disappearing (CIAT 2020). To prevent the permanent loss of sorghum wild species, collection activities are needed in regions of Africa and Australia.

### Germplasm preservation

Proper storage conditions help to ensure the optimal preservation of sorghum germplasm. The USDA NPGS (National Plant Germplasm System) sorghum collection is divided into multiple inventories. Distribution inventories are stored at 4 °C with 25% humidity while long-term inventories are stored at − 18 °C in sealed foil bags. A back-up inventory is stored at the USDA National Laboratory for Germplasm Preservation in Fort Collins, Colorado to safeguard germplasm against unforeseeable events, such as weather-related disasters, that may impact the active collection stored in Griffin, GA. A select number of accessions (~ 20%) are further safeguarded in the Svalbard Global Seed Vault in Norway.

Germplasm regenerations are required to maintain viable seed in these collections. Viability testing is conducted periodically to monitor seed health and accessions low in seed quantity or with reduced germination rates or viability are prioritized for regeneration. For the USDA collection, regenerations are conducted in Mayaguez, Puerto Rico annually as this location allows for flowering of short-day sorghum accessions. To prevent cross-pollination, the panicles are bagged prior to anthesis. During regeneration, basic descriptor data are recorded when possible and made available on the Germplasm Resources Information Network (GRIN)-Global for the USDA, NPGS collection.

### Core and mini-core collections

To facilitate the evaluation of plant germplasm collections, the core collection concept was adopted from studies in humans (Frankel 1984). A core collection can be developed for any species and typically represents 10% of the entire collection (Brown 1989a, b). Sorghum core collections have been established for the ICRISAT and USDA NPGS collections. The first ICRISAT sorghum core collection (3475 accessions) was established using seven morphological traits from 33,100 accessions (Rao et al. 1996). The second core collection (2247 accessions) was established using photoperiod-sensitivity grouping and logarithmic random sampling from 22,473 landraces at ICRISAT (Grenier et al. 2001a, b). The USDA NPGS sorghum core collection (3011 accessions) represents 77 countries and was established by Dahlberg et.al. (Dahlberg et al. 2004). The number of accessions in a core collection is greatly reduced and can be useful for large institutions, but the sheer amount of material can still prove challenging for small institutions to handle. Thus, the mini-core concept was postulated, representing 10% of the core collection or 1% of the entire germplasm collection, of a sorghum gene bank (Upadhyaya and Ortiz 2001). The first sorghum mini-core (containing 242 accessions) collection was developed using 21 morpho-agronomic traits and passport information from 2246 accessions of the core collection (Upadhyaya et al. 2009). The sorghum core and mini-core collections have been successfully used for identification of accessions with different traits of interest (Upadhyaya et al. 2016).

### Subset collections orientated by geographic locations

For purposes of identifying specific adaptive traits, evaluation of germplasm accessions collected from particular geographic locations may be the most efficient approach, because these accessions have evolved and developed adaptive traits due to selection pressure under local environments. For example, many accessions with superior resistance to Anthracnose disease were identified from a small subset of accessions from the geographic region of Mali where Anthracnose is highly prevalent (Erpelding 2012). Two subsets from the Ethiopia sorghum germplasm collections at NPGS USDA were constructed and evaluated by two independent research groups. A subset of 374 accessions from Ethiopia was phenotyped and genotyped by sequencing (GBS), and these studies revealed that the NPGS Ethiopia germplasm is comprised of 11 populations with high levels of admixture (Cuevas et al. 2017). A subset of 1,425 Ethiopia landrace accessions was also phenotyped and genotyped by sequencing, and the genetic architecture and natural variation of the Ethiopian germplasm was identified, and the associated SNPs can be used as markers for molecular breeding (Girma et al. 2019). To identify sources of resistance to anthracnose and grain mold, 158 Senegal accessions from NPGS collection of USDA were screened in two locations (Cuevas et al. 2018b). From these screenings, eight accessions were identified as resistant to both diseases and 14 accessions were identified as resistant to grain mold alone. In another study, 55 accessions resistant to anthracnose were identified from 318 accessions of Sudan germplasm curated in the NPGS core collection (Cuevas and Prom 2020).

Recently, a subset of 387 lines of Ethiopian germplasm was assembled using a comprehensive phenotypic and genomic characterization (Girma et al. 2020). First, a subset of 2010 accessions were selected from 9000 total accessions in the Ethiopia sorghum germplasm bank and phenotyped at three locations for six different quantitative traits. Then, 1628 accessions were genotyped by sequencing (GBS), and a high level of genetic diversity and rare natural variation were identified in the collection. Finally, the core subset representing the Ethiopia landrace germplasm was selected and assembled following posteriori grouping of genotypes, genetic clusters, and stratified random sampling using quantitative traits. The above studies demonstrate that core collections from geographical regions are very efficient resources for evaluation and utilization of sorghum germplasm.

## Sorghum linkage and association mapping resources

Linkage and association mapping are widely used approaches to understand the genetic basis of natural variation of plant traits (Huang and Han 2014). These approaches are used to characterize the genetic architecture of traits (number of loci, distribution of loci, gene action, linkage, and allele frequency), generate hypotheses on the genes that underlie trait variation, and test hypotheses on the conservation of trait genetics across plant species. The use of natural variation, or standing polymorphisms, for genetic studies has several strengths (Brachi et al. 2011) and can complement mutant analysis. First, natural variation has been filtered by natural and/or artificial selection, so the variants identified are more likely to have direct ecophysiological relevance and agronomic utility. Second, genetic studies of natural variation can leverage eco-geographic and historical information. Finally, genetic analysis of natural variation can illuminate evolutionary processes that led to extant patterns of variation. Given the great diversity in sorghum germplasm, it is well-suited to natural variation-based approaches (Boyles et al. 2019). Conversely, some challenges of natural variation-based (Morris et al. 2013c) approaches, such as the complexity of the germplasm or the relative subtlety of phenotypic variation, are addressed by mutant analysis.

All genetic mapping approaches depend on recombination to shuffle genomic variation, so that statistical associations of genotypic variation (i.e., markers) with phenotypic variation (i.e., traits) will occur preferentially near the causal variant. Given high-density genome-wide markers, the resolution of mapping depends primarily on the density of recombination (Korte and Farlow 2013). In linkage mapping, crosses among parents are used to generate recombination events, while historical recombination events are used in association mapping. For both approaches, immortalized inbred lines are necessary for mapping of most complex traits, to allow genotypes to be replicated, grown under contrasting environments, and grown in agronomically relevant plant stands. Sorghum is well suited for development of immortalized mapping populations because it can be both readily crossed and self-fertilized (Barnaud et al. 2008). Here, we focus on linkage and association mapping panels that are freely available to the community via the NPGS and describe the marker genotype resources associated with each panel.

## Linkage mapping resources

Many linkage mapping studies have been conducted in sorghum in the past 25 years that have investigated the genetic basis of traits, such as height (Hilley et al. 2017; Lin et al. 1995; Yamaguchi et al. 2016), flowering time (Casto et al. 2019; Guitton et al. 2018; Mace et al. 2013a), pigmentation (Wu et al. 2012; Morris et al. 2013b), drought tolerance (Hayes et al. 2016; Subudhi et al. 2000; Tuinstra et al. 1996), cold tolerance (Burow et al. 2011; Knoll et al. 2008; Marla et al. 2019), and disease resistance (Upadhyaya et al. 2013). Unfortunately, much of the germplasm and associated marker data for earlier work is not readily available in public repositories (Subudhi et al. 2000; Tuinstra et al. 1996; Wu et al. 2012). Thankfully, an increasing number of sorghum RIL families are being deposited and made easily accessible to the community via NPGS-GRIN.

In the NPGS-GRIN system, sorghum RIL families are listed under the crop designation of “SORGHUM-GENSTOCKS”. Four of these RIL families were developed by the USDA Plant Stress and Germplasm Development unit in Lubbock, Texas. The first of these, BTX623*IS3620C, is derived from a cross between the genome reference line BTx623 and the highly-divergent (and possibly independently domesticated) Nigerian guinea margaritiferum line IS3620C (Camjin) (Burow et al. 2011). Given (i) the wide divergence between the parents, (ii) the large family size, and (iii) the availability of high-density GBS markers, this population can be a powerful resource for the dissection of many traits (Kong et al. 2018). In addition, three RIL families developed to study cold tolerance, derived from crosses of cold-susceptible US parent lines with cold-tolerant Chinese parent lines, are currently available from GRIN along with GBS markers (RTx430*Gai Gaoliang, BTx623*Hong Ke Zi, BTx623*Niu Sheng Zi) (Burow et al. 2011; Marla et al. 2019). While these families were primarily developed for studies of cold tolerance, they segregate for many other traits of interest, such as pigmentation, flowering time, plant architecture, and inflorescence architecture (Marla et al. 2019). Other RIL families available from GRIN are a part of the NAM resource described below.

## Association mapping resources

With the advent of methods to generate high-density genome-wide markers, much of the focus of genetic mapping in crops has shifted from linkage mapping and candidate gene association to genome-wide association studies (GWAS) (Huang and Han 2014). For the purposes of this review, we will refer to association mapping “panels” rather than populations, to distinguish the germplasm sets from the population they were derived from. The most widely used sorghum GWAS resource is the sorghum association panel (SAP). The SAP global diversity panel with GBS marker data (Hu et al. 2019; Morris et al. 2013b) that is available from GRIN. The SAP was designed to capture sorghum’s global diversity of form, function, and end uses (Casa et al. 2008), and includes predominantly grain sorghum types, along with some sweet sorghums, forages, and broomcorns. Importantly, all SAP lines flower in temperate latitudes, either because they are temperate-adapted genotypes or because they are sorghum conversion (SC) lines, which are tropical accessions converted to photoperiod insensitivity and short stature (Klein et al. 2008; Stephens et al. 1967). Many traits have been subjects of GWAS using the SAP, as recently reviewed (Boyles et al. 2019; Mural et al. 2020).

Another global diversity panel available from GRIN is the bioenergy association panel (BAP) (Brenton et al. 2016), consisting predominantly of photoperiod-sensitive tropical accessions, along with sweet and forage sorghums. The BAP has associated SNP data from GBS and whole-genome resequencing (Bellis et al. 2020; Brenton et al. 2016; Lozano et al. 2021). A collection of over 2000 georeferenced sorghum landraces, many of which are available from GRIN, was genotyped using GBS and is available for phenotypic and environmental association studies (Bellis et al. 2020; Lasky et al. 2015; Wang et al. 2020). In addition, GBS SNP data are available for a large number of landrace accessions from Africa, many of which are georeferenced, including a core collection for Ethiopia (Cuevas et al. 2017), Sudan (Cuevas and Prom 2020), sweet sorghum (Cuevas et al. 2019b) and the entire GRIN collections from Niger (Maina et al. 2018), Nigeria (Olatoye et al. 2018), and Senegal (Faye et al. 2019).

It is important to note that these resources were developed to capture diversity, not to conform to the assumptions of association mapping (i.e., a lack of population structure), so key caveats apply to any GWAS using these association resources. The availability of genomic resources, such as panels and markers, has made GWAS a popular approach for sorghum in recent years (Boyles et al. 2019; Morris et al. 2013b); however, GWAS may or may not be effective depending on the target traits and germplasm. Because association mapping depends on historical recombination generated by uncontrolled population processes over evolutionary timescales, assortative mating and selection cause the genetic structure of association mapping panels to deviate from what is ideal for association models (Korte and Farlow 2013). Not only can these deviations lead to false positives and false negatives, but the false findings can also be misleading (i.e., more data lead to more statistically significant false results) (Platt et al. 2010).

Under some conditions, general linear models with population terms (i.e., Q) or mixed linear models with kinship terms (i.e., K or Q + K) can account for polygenic background effects and reduce false positive associations while retaining true positives. However, when oligogenic variants (i.e., QTL) are colinear with polygenic and/or neutral background variation (which is presumably common for environmentally adaptive traits) the polygenic term can account for oligogenic variation and leads to false negatives, which has been observed in sorghum (Lasky et al. 2015; Morris et al. 2013c). Given the typical focus on avoiding false positives, the possibility of false negative is arguably an underappreciated challenge of association studies in sorghum.

Interpretation of association mapping requires thorough consideration of the population and evolutionary genetics of the source germplasm, to a greater degree than linkage mapping (Brachi et al. 2011). Those interested only in generating candidate genes for fine mapping may find that a biparental linkage approach avoids the complexity and ambiguity of GWAS interpretation (Casto et al. 2019; Hilley et al. 2017). By contrast, those interested the broader patterns of diversity and the forces that generate them may find that challenges of GWAS interpretation are worth surmounting, as in some studies of sorghum global adaptation (Lasky et al. 2015; Wang et al. 2020; Wu et al. 2019).

## Multi-parent mapping resources

Given the tradeoffs between linkage and association mapping, there has been increasing interest in developing alternative genetic mapping approaches that combine the strengths linkage mapping (power, sensitivity) with those of association mapping (diversity, resolution) (Brachi et al. 2011; Korte and Farlow 2013). Several such multi-parent mapping approaches including nested association mapping (NAM), backcross NAM, and multi-parent advanced generation intercross (MAGIC), have been applied in sorghum (Boyles et al. 2019).

The NAM approach was pioneered in maize and now used in many crops, and it uses multiple RIL families that have diverse founders but share a common parent (Gage et al. 2020; Yu et al. 2008). A global grain sorghum NAM resource is available in sorghum, developed using RTx430 as the common founder line and 10 diverse global founders, for a total of > 2200 RILs in 10 families (Bouchet et al. 2017; Yu et al. 2013). RTx430 was selected as the common parent because it has been the most important public pollinator line in recent decades (Miller 1984; Smith and Frederiksen 2000). Importantly, all the NAM parents are a part of the SAP (Yu et al. 2013), and this overlap of germplasm between these mapping resources facilitates the cross-comparison and validation of findings between NAM and SAP studies (Olatoye et al. 2020b). SNP markers for the sorghum NAM were originally developed using the v.2 BTx623 reference genome (Bouchet et al. 2017), but an updated version of the marker data are now available based on the v.3 BTx623 reference genome (Hu et al. 2019). This NAM resource has been used to map flowering time, height, inflorescence morphology, and vegetative morphology (Bouchet et al. 2017; Hu et al. 2019; Olatoye et al. 2020a, b). Notably, it has been demonstrated that the NAM resource has greater power to map some adaptive traits that would be confounded by population structure in GWAS (Bouchet et al. 2017; Olatoye et al. 2020b).

Another multi-parent mapping approach that balances some tradeoffs of linkage and association mapping is MAGIC (Huang et al. 2015). Like NAM, MAGIC seeks to increase allelic diversity compared to biparental linkage families while increasing power and specificity of QTL detection relative to GWAS. Unlike NAM, however, MAGIC aims to increase recombination and mapping resolution using advanced intercrosses and to balance allele frequencies using equal contributions from all founder lines. To date, one sorghum MAGIC resource has been described, which can be requested from the authors that developed it (Ongom and Ejeta 2018). The backcross NAM (BCNAM) approach, which uses backcrossing and selection to recover lines that are phenotypically similar to the elite common parent, can have the advantage of greater agronomic and breeding relevance compared to NAM (Jordan et al. 2011).

## Future mapping resources needed

The rapid advances in sorghum mapping resources in recent years has certainly facilitated various genetic studies. However, there are two notable gaps that remain. First, causative variants underlying sorghum natural variation have only been identified for a few traits (Casto et al. 2019; Hilley et al. 2017; Wu et al. 2019; Zhang et al. 2018; Zou et al. 2020). Generally, sorghum mapping studies have identified QTL and suggested candidate genes but have not identified the causative variants. Future resource development should concentrate on facilitating identification of causative variants (as opposed to capturing additional diversity). For instance, high-quality whole-genome resequencing or de novo genome sequencing of the NAM parents and the SAP could facilitate the discovery of causative variants from sorghum NAM and SAP-GWAS studies. The second major gap that exists is that there are cases where genetic mapping has led directly to advances in sorghum improvement. This issue is not unique to sorghum (Bernardo 2016), but future efforts may need to focus on approaches designed for direct relevance of sorghum improvement. For example, future initiatives to develop mapping resources may have a greater impact on sorghum improvement if they focus on BCNAM or other approaches that put diversity in the context of elite genetic backgrounds.

## Resources in plant dieses resistance

The resilience and adaptability of sorghum to multiple agriculture systems located in the tropical, sub-tropical, and temperate regions of the world result in the crop facing challenges from several different biotic constraints. The different end uses of the crop for food, animal feed, forage, and bioenergy determine the relevance of each disease. Sorghum productivity and profitability worldwide are limited by several diseases, such as anthracnose (*Colletotrichum sublineolum*), stalk rot (*Fusarium tapsinum* and *Macrophomina phaseolina*), head smut (*Sporisorium reilianum*), downy mildew (*Peronosclerospora sorghi*), rust (*Puccinia pupurea*), leaf blight (*Exserohilum turcicum*), and grain mold (multiple fungal species), among others, which reduce biomass and seed yield and quality.

Currently, the preferred strategy to control sorghum diseases is the identification and incorporation of resistance genes. The narrow genetic diversity among improved sorghum varieties and temperate-adapted germplasm predisposes the crop to a devastating epidemic. Over the last 30 years, multiple disease resistant accessions have been identified that are adapted to tropical and temperate climates; however, the genetic control underlying these resistance responses is unknown for most of these accessions. The recent genomics and germplasm resources available for the crop are providing insights to the genetic control of most important diseases and the molecular tools for the effective use of resistance germplasm through marker-assisted selection.

### Disease resistance germplasm in diversity panels

The two most widely used sorghum association panels used to study disease resistance responses are the SAP (Casa et al. 2008) and the ICRISAT mini-core collection (Upadhyaya et al. 2009). Both association panels enclose a large degree of genetic diversity and are genetically characterized based on GBS marker data (Lasky et al. 2015; Morris et al. 2013a) providing the research tools to elucidate disease resistance responses.

Stalk rots, a disease that causes significant damage to root and stalk tissue, was the first one to be studied with the SAP (Adeyanju et al. 2016). The GWAS based on the stalk rots resistance response of 300 accessions and 79,132 SNPs found 14 loci in chromosome 2, 3, 4, 7, 8, and 9. Remarkably, a 2-Mb genomic region at chromosome 9 is associated with stalk rots resistant across multiple environments and explains up to 16% of the observed variation. Anthracnose is a disease that affects both grain and biomass and causes significant yield losses in humid production regions (Thakur and Mathur 2000). The analysis of the disease resistance response of the SAP in Puerto Rico, Georgia, U.S. and Texas, U.S. (Prom et al. 2019; Cuevas et al. 2018a) identified 40 resistant accessions across locations. These resistance accessions clustered within different sorghum races, suggesting the presence of multiple resistance sources. Genome-wide association analysis based on binary resistance response (i.e., resistant and susceptible) identified three loci at the distal region of chromosome 5 (Cuevas et al. 2018b). These three loci explained only a limited portion of the phenotypic variation, indicating the presence of other resistance sources that have not been detected due to their low frequency or due to an overcorrection for population structure. Grain mold is a disease caused by multiple pathogenic and opportunistic fungi that affect the plant from anthesis to harvest and reduce the grain yield and quality (Bandyopadhyay et al. 2000). The SAP was evaluated for grain mold resistance in Puerto Rico (Cuevas et al. 2019a) and Texas, U.S. (Prom et al. 2020) over three consecutive years and 18 and 3 highly resistance accessions were identified, respectively. Three loci in chromosomes 1, 8, and 10 were associated to resistance response in Puerto Rico, while in Texas, U.S. multiple candidate genes were identified based on the top 10 significance SNPs. Pathogens and environments differing among both locations had large effects upon the resistance response and the GWAS results. These GWAS studies demonstrated the efficacy of SAP for the genomic dissection of disease resistance response but also showed the necessity to use other family-based approaches.

The ICRISAT mini-core collection was first evaluated for grain mold and downy mildew resistance response in India (Sharma et al. 2010). This two-year screening identified 50 and six accessions resistance to grain mold and downy mildew, respectively. Likewise, the evaluation for resistance to anthracnose, leaf blight and rust found that 13, 27, and 6 accessions exhibited resistance response to these diseases, respectively (Sharma et al. 2012). A subsequent GWAS analysis using 14,739 SNPs and the anthracnose resistance response identified 8 resistance loci and several candidate genes related to the plant immune system (Upadhyaya et al. 2013). The mini-core was also evaluated for anthracnose, downy mildew, and head smut resistance response against multiple U.S. isolates from each disease in greenhouse (Ahn et al. 2019). Nevertheless, the GWAS analysis could not found significant associations with the variation in disease resistance. Most of the accessions in the ICRISAT mini-core are originally from tropical regions; therefore, its evaluation in temperate regions might be limited to greenhouse studies due to its photoperiod sensitivity.

### Disease resistance germplasm in ex-situ collection

The narrow genetic diversity among temperate-adapted and breeding germplasm encourages the search for new disease resistance sources in tropical germplasm collections. Over the last six years, GBS techniques have been used to genetically characterize collections from Ethiopia, Sudan, Nigeria, and Senegal (Cuevas et al. 2017; Cuevas and Prom 2020; Girma et al. 2019; Olatoye et al. 2018; Faye et al. 2019). Mapping Anthracnose resistance in two core sets from U.S. NPGS Ethiopian and Sudan germplasm collection identified two resistance loci in chromosome 9 and 5, respectively (Cuevas et al. 2019b; Cuevas and Prom 2020). These two loci explain a limited portion of the observed phenotypic variation, and both resistance alleles are present in the SAP. A large GWAS analysis for grain mold resistance using 1425 Ethiopian landraces from the Ethiopian Biodiversity Institute identified a major locus at chromosome 1 (Nida et al. 2019) that was associated with the biosynthetic pathway of flavonoid compounds in the seeds. Gene expression analysis of candidate genes was based on resistant and susceptible temperate-adapted germplasm; thus, this resistant allele is also present in the SAP. While multiple disease resistance sources have been identified in tropical sorghum germplasm, further research efforts are needed to identify which resistance alleles are present in temperate-adapted and breeding germplasm. Realizing the untapped potential of new resistance alleles in tropical germplasm will require the development of segregating mapping populations using multiple genetically diverse resistance sources to overcome the limitations of the GWAS analysis.

### Challenges and further breeding approaches

Multiple disease resistance sources are present in tropical and temperate-adapted germplasm. The genomic resources in both groups of germplasm encourage screening to identify and delimit genomic regions associated with disease resistance responses. Elucidation of the disease resistance sources present in temperate-adapted germplasm is necessary to confirm the identification of new sources in tropical germplasm. In parallel, the screening of tropical germplasm could be focused on new sources of resistance or alleles that could also enhance the genetic diversity of temperate-adapted germplasm. The creation and use of core sets of tropical germplasm might help to reduce the number of accessions that need to be evaluated for disease resistance in the greenhouse and field. Similarly, the improvement and development of new disease screening assays based on *omics* technology (e.g., phenomics and metabolomics) will be relevant for the identification of resistance germplasm.

## Sorghum hybrid seeds production

The production of sorghum hybrid seed relies on the cytoplasmic male sterility system (CMS). Three different CMS systems have been identified in sorghum, but the original system, designated as A1, is the most commonly used system for producing commercial sorghum hybrids (Kuhlman et al. 2006). The CMS breeding system consist of three lines and two parental groups (A/B and R). The A/B parental group involves the female parent, or A line (cytoplasm male-sterile), which is pollinated by its isocytoplasmic maintainer, or B line (normal cytoplasm), to regenerate seeds of the A line (i.e., two lines to maintain cytoplasm male sterility). The R parental group includes the restorer, or R lines, that are used to produce fertile progeny by its cross to the female parent A line (Rooney 2004). Hence, heterosis in sorghum is determined by the complementary genes among the A/B-lines (female) and R lines (restorer/male) parental pools in breeding programs (Mindaye et al. 2015). Genetic studies based upon molecular markers show that the genetic diversity in the R parental gene pool is significantly larger than in the A/B parental gene pool (Menz et al. 2004; Silva et al. 2021). Evidently, this imbalance in diversity between these two parental groups constrains the development of higher-yielding sorghum hybrids.

Broadening the genetic base of the A/B parental gene pool requires the identification of tropical germplasm that maintains the cytoplasm male sterility. This assignment is determined by testcrossing tropical germplasm to the A line to evaluate the fertility of the F_1_ progeny. When the F_1_ progeny is male-sterile, the tropical accession is classified as a maintainer (i.e., B line) and assigned to the A/B parental gene pool. In addition to the low frequency of maintainer lines among temperate-adapted and tropical germplasm (Madugula et al. 2018), new maintainer lines also need to be able of produce F_1_ progeny male-sterile under broad environments where sorghum breeding is possible. Today, less than 2% of the NPGS tropical germplasm has been classified into the A/B parental gene pools. The integration of multiple *omics* techniques may provide insight into the phenomena of CMS and fertility restoration in crops (Bohra et al. 2016). Nevertheless, it is imperative for sorghum breeding programs to identify additional germplasm belonging to the A/B parental pool to construct favorable heterotic groups that permit the creation of superior hybrids.

## Perspectives

New sequencing technologies are continuously developed that achieve higher throughputs with lower associated costs. It is now already possible to map and identify causal genes represented by interesting mutants using BSAseq, and it will soon become routine to rapidly identify causative mutations using the BSAseq workflow that is freely available online. Several SAPs have been established, and it is now feasible to sequence every line in the SAP, producing high density and high quality of DNA markers and greatly enhancing the power of mapping the causative genes under QTL of agronomic traits. In the years ahead, the sequenced pedigreed mutant libraries will be crucial to validate the causative genes under QTL. The rapid increase in genomic data will be integrated online in Sorghumbase (Gladman et al. 2021 in this collection issue), providing a valuable resource to accelerate breeding throughput. Mutant libraries, QTL mapping through SAP, and biparental mapping will lead to the discovery of many causative genes and provide valuable potential targets for genome editing. These rapid advances in many frontiers of sorghum research make it impractical, if not impossible, for one laboratory to perfect and specialize in all areas of research. It is therefore certain that a true revolution in sorghum breeding will rely on the close collaboration of the entire global sorghum community.

### *Author contribution statement*

ZX conceived the idea. All authors participate the writing and agree with the final draft.

## Data Availability

Dataset(s) derived from public resources and made available with the article.
